# The association between Salt-inducible kinase 2 (SIK2) and gamma isoform of the regulatory subunit B55 of PP2A (B55gamma) contributes to the survival of glioma cells under glucose depletion through inhibiting the phosphorylation of S6K

**DOI:** 10.1186/s12935-015-0164-6

**Published:** 2015-02-18

**Authors:** Ya-nan Li, Yi-qun Cao, Xi Wu, Guo-sheng Han, Lai-xing Wang, Yu-hui Zhang, Xin Chen, Bin Hao, Zhi-jian Yue, Jian-min Liu

**Affiliations:** Department of Neurosurgery, Changhai Hospital, 168 Changhai Road, Shanghai, 200433 China

**Keywords:** PPP2R2C, glucose metabolism, Glioma cell, B55gamma, SIK2

## Abstract

**Background:**

PPP2R2C encodes a gamma isoform of the regulatory subunit B55 subfamily consisting PP2A heterotrimeric with A and C subunits. Currently, the precise functions of B55gamma in cancer are still under investigating. In this project, we reported a novel function of B55gamma in the regulation of glucose metabolism in Glioma cells.

**Methods:**

Western blot and immunoprecipitation were performed to determine protein expression and interaction. Cell viability was measured by Typan Blue staining and direct cell counting using hematocytometer. siRNA technology was used to down regulate protein expression.

**Results:**

Glucose uptake and lactate product were suppressed by overexpression of B55gamma in Glioma cells. In addition, cancer cells with larger amount of B55gamma showed higher survival advantages in response to glucose starvation through the dephosphorylation of S6K. From proteomic analysis, we found B55gamma binds with and up regulates SIK2 through the stabilization of SIK2 protein which is required for the B55gamma-mediated suppression of S6K pathway. Knocking down of SIK2 in B55gamma over expressing cells recovered the phosphorylation of S6K.

**Conclusion:**

In summary, our project will provide novel insight into the design and development of therapeutic strategies to target the B55gamma-mediated glucose metabolism for the treatment of human brain tumor patients.

## Introduction

The PP2A heterotrimeric protein phosphatase is a ubiquitous and conserved serine/threonine phosphatase with broad substrate specificity and diverse cellular functions [1]. It is composed of one structural A, one catalytic C, and one regulatory B subunit that associates with a variety of substrates [2]. Among the targets of PP2A are proteins of oncogenic signaling cascades, such as Raf [3], MEK [4], and AKT [5]. This suggests that specific PP2A holoenzymes play a role in cellular proliferation and oncogenic transformation [2]. The regulatory B subunit can modulate the activity of the PP2A, by targeting a wide range of PP2A substrates. Four unrelated families of B subunits have identified to date: B/B55/PR55/PPP2R2, B0/B56/PR61/PPP2R5, B/PR72/PPP2R3, and Striatin [6]. PPP2R2C encodes a gamma isoform of the regulatory subunit B55 subfamily-B55 gamma. Currently, the precise functions of B55gamma are still under investigating. It has been reported that B55gamma was an inhibitor of c-Jun NH2-terminal kinase (JNK) activation by UV irradiation [7]. Currently, the function of B55gamma in tumor progression has not been well studied yet. A recent paper claimed that B55gamma might be a tumor suppressor gene in prostate cancers, they reported the loss of B55gamma promotes androgen ligand depletion-resistant prostate cancer growth, which was independent of AR-mediated transcriptional programs [8].

The Warburg effect is the observation that most cancer cells predominantly produce energy by a high rate of glycolysis followed by lactic acid fermentation in the cytosol, rather than by a comparatively low rate of glycolysis followed by oxidation of pyruvate in mitochondria as in most normal cells [9]. Mutations in oncogenes and tumor suppressor genes are known to be responsible for malignant transformation, and the Warburg effect is the result of oncogenic stimulation and loss of tumor suppressor genes that lead to a series of metabolic reprogramming, such as the expression and/or translocation of glucose transporters to the plasma membrane [9,10] and upregulation or activation of glycolytic enzymes. So far, multiple oncogenes or tumor suppressors have been reported to participate into the regulatory pathways in cancer cells glucose metabolism, such as AKT [11], PI3K [12], P53 [13], PP2A [14], src [15], Ras [16] and c-myc [17].

Salt-inducible kinase (SIK1–3) is a serine/threonine protein kinase which belongs to a family of AMP-activated protein kinases (AMPKs) [18]. SIK2 has been shown to function in the insulin-signaling pathway during adipocyte differentiation and to modulate CREB-mediated gene expression in response to hormones and nutrients [19]. However, molecular mechanism underlying the regulation of SIK2 kinase activity remains largely elusive.

In this study, we reported a novel function of B55gamma in the regulation of glucose metabolism in Glioma cells. Exogenous over expression of the B55gamma in Glioma cells suppressed the cancer cell glucose uptake and lactate product, indicating a putative tumor suppression role. Glioma cells with larger amount of B55gamma showed higher survival advantages in response to glucose deprivation through the inhibition of S6K pathway. Furthermore, from proteomic analysis, we found B55gamma binds with SIK2 and increases SIK2 stability in Glioma cells which is required in the B55gamma-mediated suppression of the phosphorylation of S6K. Knocking down of SIK2 by siRNA recovered the phosphorylation of S6K. In summary, our project will provide novel insight into the tumor suppression function of B55gamma in the regulation of glucose metabolism in Glioma cells. Our study will benefit the development of therapeutic strategies for targeting this gene to treat human brain tumor patients.

## Materials and methods

### Cell culture and antibodies

Human glioma cell lines U251, U138, LN229 and Normal human astrocyte (NHA) were purchased from American Type Culture Collection (ATCC). Cells were grown adherently in DMEM media supplemented with 10% fetal bovine serum (FBS; Sigma-Aldrich Chemical Company) and 1% penicillin-streptomycin (GIBCO BRL, Grand Island, NY) and maintained in a humidified incubator containing 5% CO_2_ at 37°C. Cells were maintained in these culture conditions for all experiments.

Antibodies used from this project were purchased from: B55Gamma (Santa Cruz: sc-100417); mTOR pathway and substrate antibody sampler kit (Cell Signaling #9862&#9964); β-actin (Cell Signaling #4967); SIK2 (Cell Signaling #6919); GLUT1 (Santa Cruz: sc-7903); HKII (Santa Cruz: sc-130358) and LDHA (Cell signaling #2012).

### Immunoprecipitation

Immunoprecipitation was conducted from cells lysed in RIPA buffer. Equal amounts of total protein were used for IP or IB. Clarified lysates (250 μg) were incubated with primary antibody and control IgG for overnight at 4°C cold room. After 2 h of incubation with agarose G plus beads, beads were washed and eluted then the elution were subjected to 10% SDS PAGE gel electrophoreses.

### Plasmid DNA and siRNA transfections

The specific siRNA to SIK2 and Control siRNA were purchased from Santa Cruz (sc-44364). Vector containing GFP-tagged ORF clone of Homo sapiens protein B55gamma, WT S6K or KA S6K were purchased from Origene. Cells were transfected for 4 hrs with 50 nM siRNA or control siRNA using Oligofectamine (Invitrogen, Carlsbad, CA, USA) according to manufacturer’s instructions. The overexpression vector containing wild-type B55gamma (2 μg), wild-type S6K (2 μg), kinase active S6K (2 μg) or control vector (2 μg) were transfected using Oligofectamine (Invitrogen, Carlsbad, CA, USA) according to manufacturer’s instructions. After transfection cells were cultured in complete medium for an additional 48 h in complete medium, and then they were processed for further analysis.

### Protein stability assay

Cells were treated with or without CHX at 100 ug/ml or MG-132 at 5 uM and collected at indicated time points. Cell lysates were collected for western blot analysis probed with anti-SIK2 antibody.

### Cell viability assay

A total of 5 × 10^4^ ~ 1 × 10^5^ cells/well were seeded in 6-well plates. Twenty-four hours later, the medium was replaced with fresh medium with or without glucose and incubated for 12, 24, 36 or 48 hrs, respectively. Cell viability was determined by Typan Blue staining and direct cell counting using hematocytometer.

### Glucose uptake and lactate production assay

Cells were seeded in 12-well plates at 1 × 10^5^ to 3 × 10^5^ cells per well. Culture media was collected at 48 hours and stored at −20 degree until assayed. Glucose uptake was measured using an Amplex Red Glucose/Glucose Oxidase assay kit (Molecular Probes). Absorbance was measured at 563 nm using a SpectraMax M5 plate reader (Molecular Devices) and the results were normalized to the amount of total protein compared with the control cells. Lactate production in the medium was detected by using a Lactate assay kit (BioVision). Results were normalized to the amount of total protein compared with the control cells.

### Western blot analysis

Cells or homogenized cortical tissue samples were prepared in NP-40 lysis buffer (150 mM NaCl, 50 mM Tris–HCl, 1% NP-40 for cell culture; 50 mM Tris, 1 mM EDTA, 5 mM MgCl2, 1% Triton X-100 for brain tissue) containing protease and phosphatase inhibitors (Roche Diagnostics GmbH) and resolved in 10 or 12% SDS gels. Proteins were transferred onto nitrocellulose membranes (Bio-Rad) and incubated with primary antibodies followed by incubation with horseradish peroxidase-conjugated secondary antibodies (GE Healthcare). The immunoreactivity was detected with an Immobilon Western Chemiluminescent HRP Substrate (Millipore).

### Statistical data analysis

All data were analysed using GraphPad Prism 5.04. The unpaired Student's *t*-test was used for the data analysis. All data were shown as mean ± standard error (SE). A statistical difference of *P* < 0.05 was considered significant.

## Results

### Overexpression of B55gamma suppresses glucose metabolism in Glioma cells

**Figure 1:**
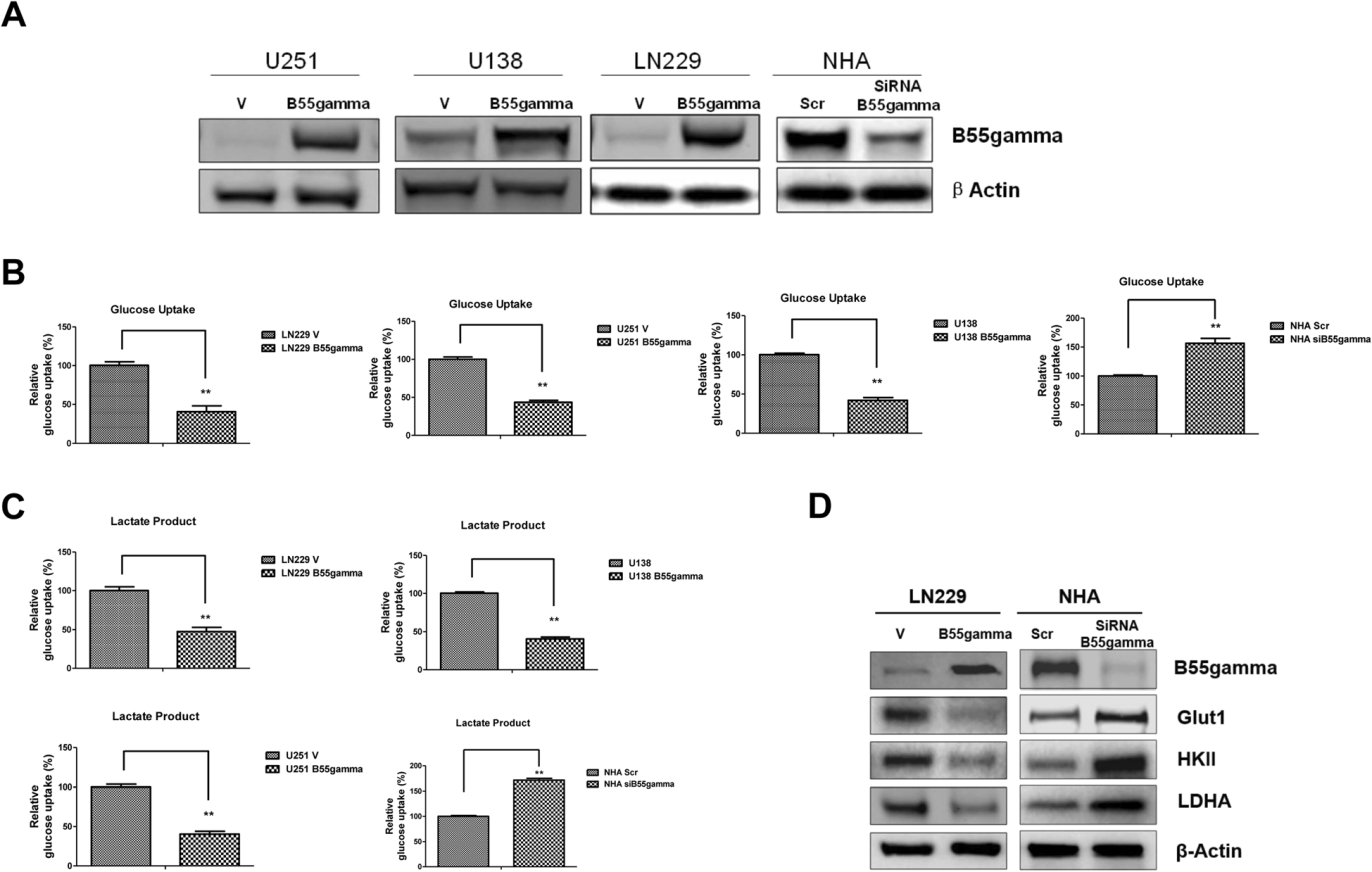
**B55gamma suppresses glucose metabolisms in Glioma cells. A**. Overexpression and knockdown of PPP2R2C encoded protein-B55gamma in U251, U138, LN229 and NHA cells. Western blot were performed with an anti-B55gamma antibody of total cell extract. β-Actin was a loading control. **B**. The over-expression of B55gamma inhibited glucose uptake in U251B55gamma, LN229B55gamma, U138B55gamma cells and knockdown of B55gamma in NHA cells increased glucose uptake compared with control cells. **C**. The overexpression of B55gamma inhibited lactate product in U251B55gamma, LN229B55gamma, U138B55gamma cells and knockdown of B55gamma in NHA cells increased lactate product compared with control cells. **D**. Overexpression of B55Gamma in LN229 cells upregulated major enzymes in glucose metabolism: Glut1, HKII, and LDHA. Knockdown of B55gamma in NHA cells downregulated Glut1, HKII and LDHA expression. Western blot were performed with an anti-Glut1, anti-HKII and anti-LDHA antibodies of total cell extract. β-Actin was a loading control. Columns, mean of three independent experiments; bars, SE. **, P < 0.01.

**Figure 2:**
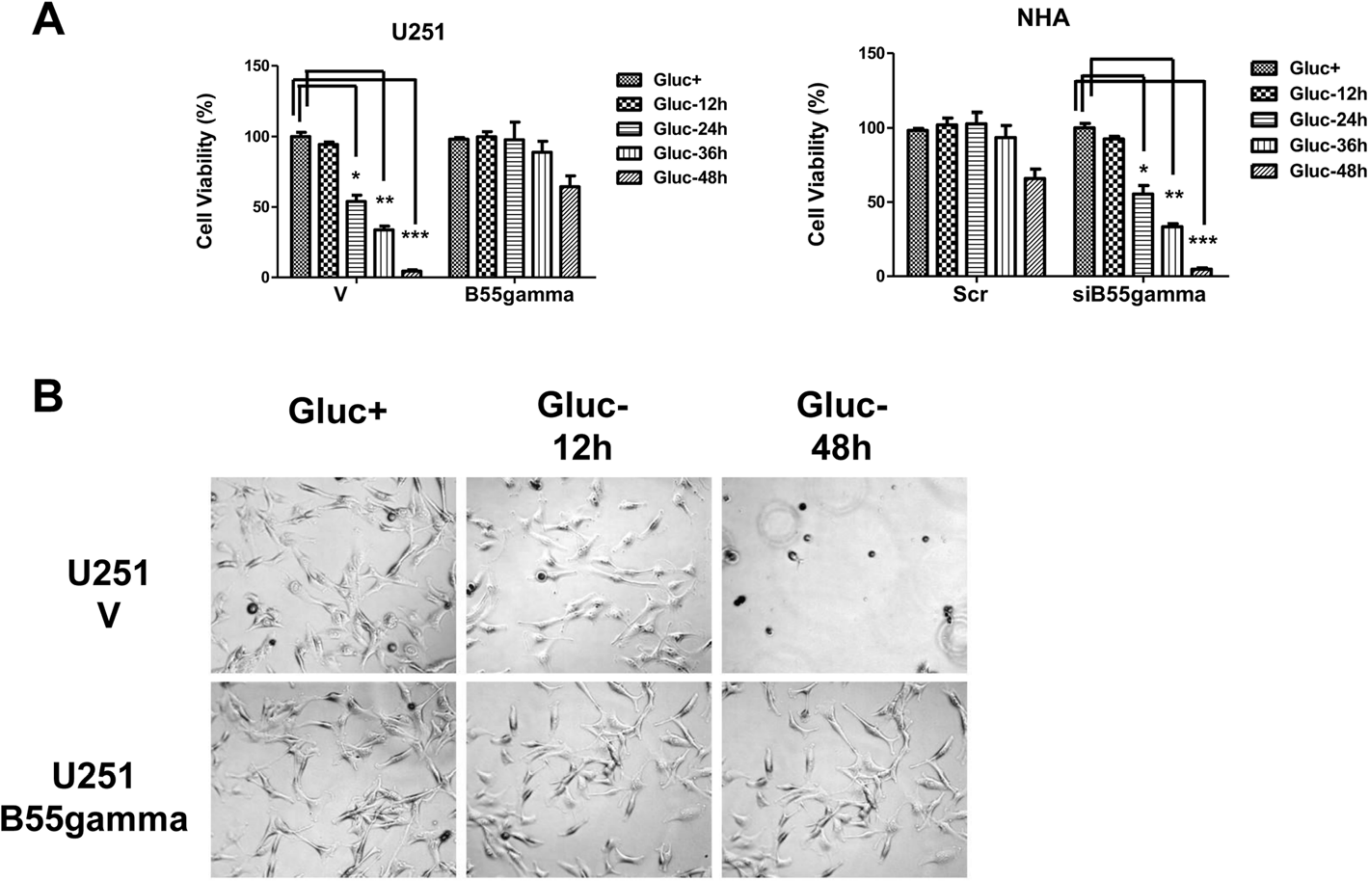
**Glioma cells with overexpression of B55gamma are insensitive to glucose starvation. A**. Overexpression of B55gamma in U87, U135 and U118 glioma cells. Glucose withdrawal induced cell death in U251V cells but U251B55gamma cells were insensitive to glucose withdrawal. Cells were starved of glucose for 12 h, 24 h, 36 h and 48 h and cell viability assay were performed by direct counting of Trypan blue negative cells. **B**. Cell death and morphological changes in U251V and U251B55gamma cells in response to glucose starvation for 12 hrs and 48 hrs. Columns, mean of three independent experiments; bars, SE. *, P < 0.05; **, P < 0.01; ***, P < 0.001.

Since B55gamma has been reported as a putative tumor suppressor gene by Bluemn EG *et al.* [9], we raised interests in the exploration of whether B55gamma regulates glucose metabolism in cancer cells. We stably transfected plasmid containing wild type B55gamma into multiple Glioma cell lines which express low levels of B55gamma originally and specifically knocked down B55gamma expression by siRNA in NHA cells which express relative higher level of B55gamma (Figure 1A). We next measured the glucose uptake and lactate product in B55gamma overexpressed cells compared with control cells. As we expected, overexpression of B55gamma in Glioma cells significantly inhibited glucose uptake and lactate product while knockdown of B55gamma in NHA cells increased glucose metabolism (Figure 2B & 2C). Consistent with our results, the major enzymes involved in the glucose metabolism were significantly down regulated in LN229 B55gamma cells and upregulated in NHA B55gamma knocking down cells (Figure 1D). Taken together, our data revealed that B55gamma plays an essential role in the inhibition of glucose metabolism in Glioma cells.

### Glioma cells with higher amount of B55gamma exhibit survival advantages in glucose starvation

We next examined whether the alteration of glucose metabolism regulated by B55gamma contributes to the toleration glucose starvation. Overexpression of B55gamma in U251 Glioma cells significantly increased the cell viability in response to glucose depletion in multiple time points compared with U251 control cells, while knocking down of B55gamma in NHA cells rendered cells sensitive to glucose depletion (Figure 2A & 2B), suggesting B55gamma participated in the regulation of cell apoptosis pathway in response to glucose starvation.

### Overexpression of B55gamma in Glioma cells inhibits S6K phosphorylation

**Figure 3:**
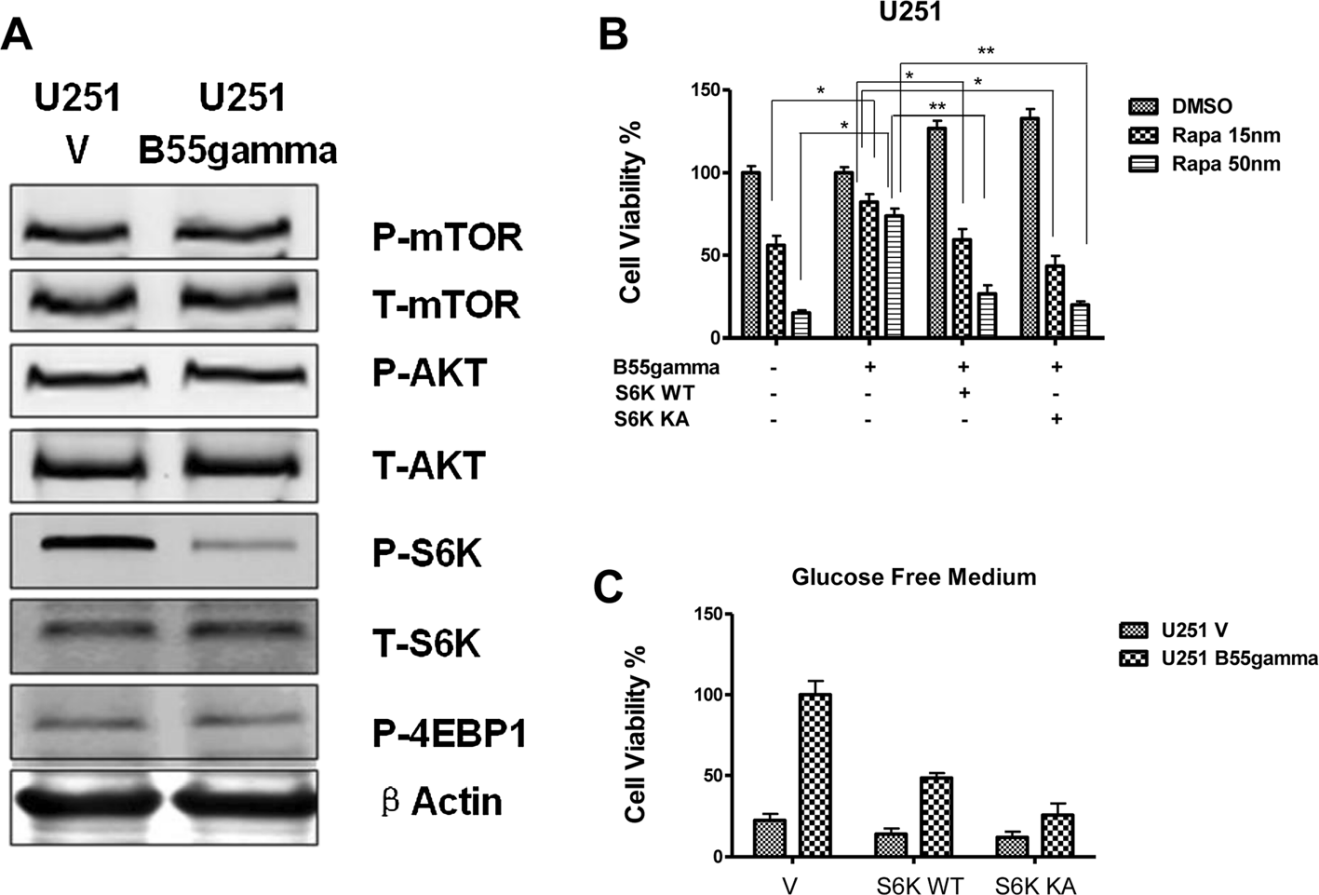
**Overexpression of B55gamma in Glioma cells inhibits the phosphorylation of S6K. A**. Overexpression of B55gamma in U251 decreases phosphorylation of S6K, but phosphorylation of 4EBP1 and mTOR and AKT have no significant change. **B**. U251 cells were transfected with control vector or B55gamma alone or B55gamma + S6K WT or B55gamma + S6K KA for 48 hrs. Cell viability assays were performed under the treatments of Rapamycin at 15 nM and 50 nM, DMSO treatment was control. **C**. Transfection of S6K WT and S6K KA into U251V and U251B55gamma cells followed by the measurements of cell viabilities under the glucose withdrawal for 24 hrs. Columns, mean of three independent experiments; bars, SE. **, P < 0.01; ***, P < 0.001.

To understand the mechanisms how B55gamma regulated cell viability, we screened multiple cell survival/proliferation pathways. Among them, we found overexpression of B55gamma significantly decreased the phosphorylation of S6K, but phosphorylation of 4EBP1 which was another substrate of mTOR complex1 did not change (Figure 3A). The phosphorylation of mTOR and upstream signaling-phosphorylation of AKT did not change (Figure 3A), indicating S6K might be a downstream target of B55gamma. To further strengthen our results, we performed experiments to check whether the B55gamma-overeexpressed phenotype can be rescued by overexpression of wild-type S6K or kinas active S6K. Our data showed U251B55gamma cells displayed more resistance to Rapamycin at different concentrations compared with U251V cells. However, restoration of S6K by transfection of wild-type S6K or kinase active S6K into B55gamma overexpressing cells re-sensitized U251B55gamma cells to Rapamycin (Figure 3B), revealing the B55gamma-mediated resistance to rapamycin and glucose depletion might be through the suppression of S6K phosphorylation. Since we hypothesized that the B55gamma-mediated inhibition of S6K activity contributed to the tolerance to glucose depletion in Glioma cells, we next tried to figure out whether exogenously expression of S6K in Glioma cell can potentially reverse the resistance to glucose depletion in U251B55gamma cells. Under glucose depletion, overexpression of S6K WT or S6K KA in U251B55gamma cells significantly decreased the cell viabilities (Figure 3C), suggesting the reduced activity of S6K might be the mechanism for the B55gamma-mediated tolerance to glucose starvation conditions.

### B55gamma interacts with SIK2 and increases the stability of SIK2

**Figure 4:**
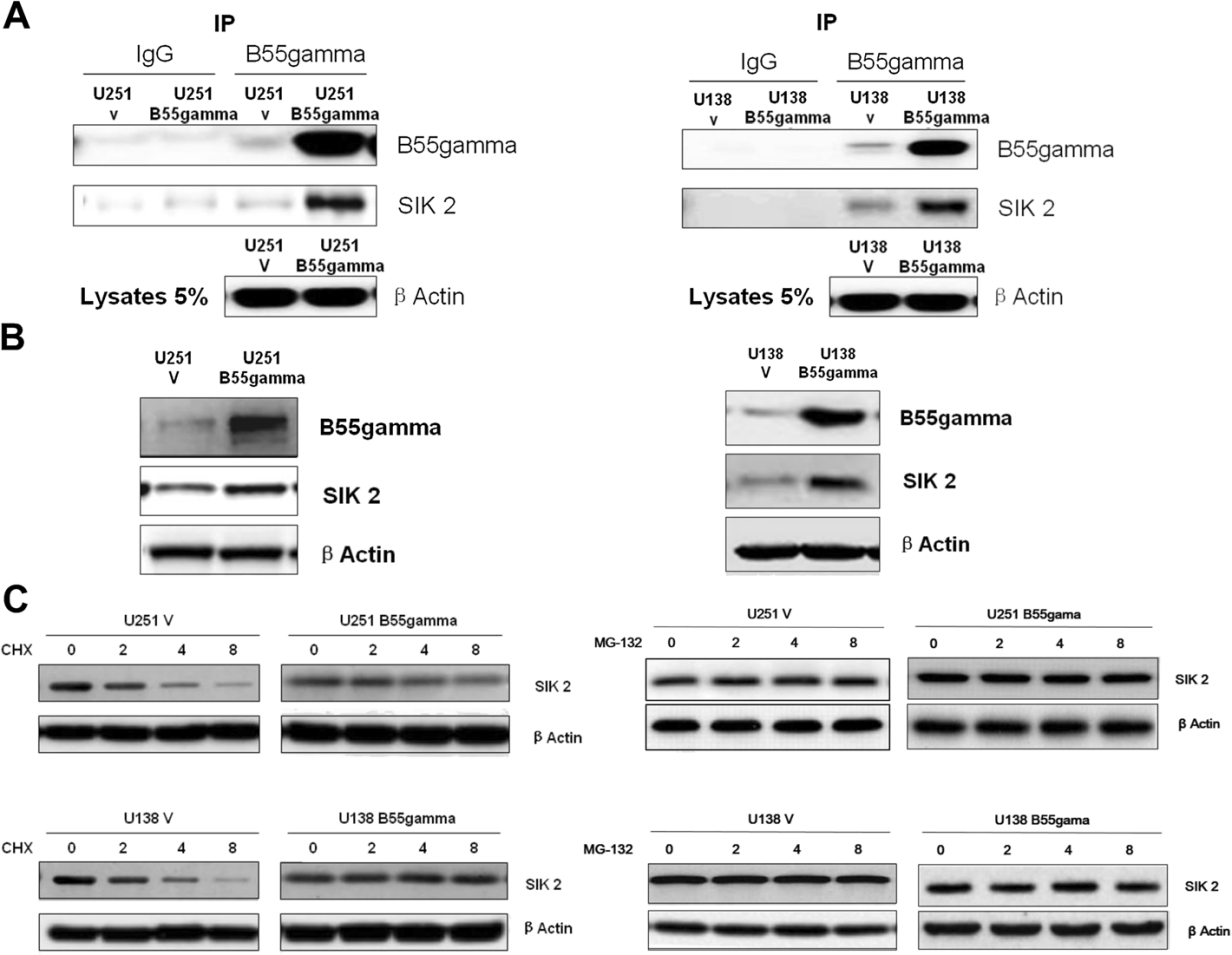
**B55gamma binds with SIK2 and increases protein stability of SIK2. A**. B55gamma interacts with SIK2 in Glioma cells. Co-Immunoprecipitation was performed using B55gamma antibody and mouse IgG as control. Immunoblotting with SIK2 antibody showed B55gamma associated with SIK2. β-Actin expression in 5% whole cell lysates were showed as loading controls. **B**. Overexpression of B55gamma in U251 and U138 cells upregulated SIK expression. Western blot were performed with an anti-SIK2 and anti-B55gamma antibodies of total cell extract. β-Actin was a loading control. **C**. U251V and U251B55gamma (upper) cells were treated with CHX at 100 ug/ml or MG-132 at 5 uM for 0, 2, 4 and 8 hrs followed by Western blotting assay to exam the protein expression level of SIK2. U138V and U138B55gamma (lower) were treated with CHX at 100 ug/ml or MG-132 at 5 uM for 0, 2, 4 and 8 hrs followed by Western blotting assay to exam the protein expression level of SIK2.

To figure out the mechanism how B55gamma regulated the dephosphorylation of S6K, we performed immunoprecipitation analysis. From the multiple putative binding partners of B55gamma, we focused on the Salt-Induced Kinase 2 (SIK2) which specifically binds with B55gamma in both U251gamma and U138B55gamma cells (Figure 4A). It was interesting that the expression levels of SIK2 were upregulated in B55gamma-overexpressing cells (Figure 4B) which pushed us forward to explore the mechanisms how B55gamma upregulated SIK2. We treated U251V and U251B55gamma cells with Cycloheximide (CHX), which inhibits protein translation in eukaryotes. Our results showed the SIK2 expression were significantly decreased when we inhibited the SIK2 protein synthesis in U251V and U138V cells. However, the SIK2 levels did not decrease in B55gamma-overexpressing cells (Figure 4C left), indicating when the protein synthesis was inhibited, B55gamma increased the stability of SIK2 to prevent degradation. In addition, our data showed the protein levels of SIK2 did not change by the treatments of MG-132 which is an inhibitor of protein degradation at 0, 2, 4 and 8 hours in both control cells and B55gamma overexpressing cells (Figure 4C right). Combined with the CHX experiments, these data showed B55gamma interacted with SIK2 and upregulated SIK2 expression level through the enhancement of protein stability.

### SIK2 is required in the B55gamma-mediated suppression of S6K phosphorylation

**Figure 5:**
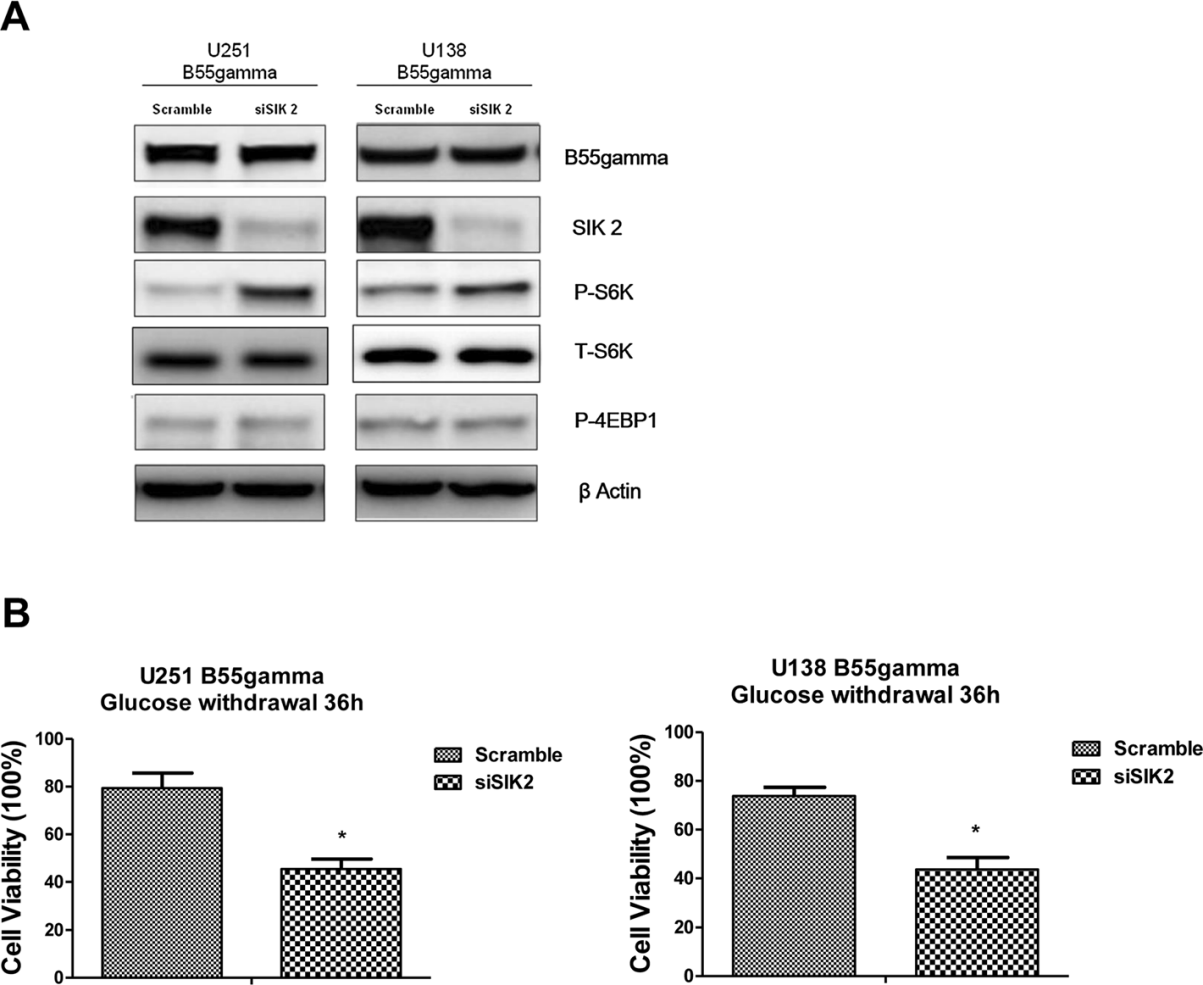
**Knockdown of SIK in B55gamma overexpression cells recovers the phosphorylation of S6K. A**. U251V, U251B55gamma, U138V and U138B55gamma cells were transfected with scramble siRNA (Ctr) or SIK2 siRNA. 48 hrs after siRNA transfection, cell lysates were prepared and Western blotting was performed to detect the phosphorylation of S6K statues. The β-actin protein was used as a loading control. **B**. U251B55gamma (left) and U138B55gamma (right) cells were transfected with scramble siRNA (Ctr) or SIK2 siRNA. 48 hrs after siRNA transfection, cell were plated into 6-well plate for overnight, then the regular medium was replaced by glucose free medium for 36 h-starvation followed by the cell viability assay. Columns, mean of three independent experiments; bars, SE. *, P < 0.05.

We finally examined whether the association of B55gamma and SIK2 is involved in the event of the deposphorylation of S6K by B55gamma. Our data showed knocking down of SIK2 significantly recovered the phosphorylation of S6K in U251B55gamma and U138B55gamma cells (Figure 5A) and the phosphorylation of 4EBP1 did not change, indicating B55gamma inhibited phosphorylation of S6K through the interaction of SIK2. We next checked the sensibilities of B55gamma overexpressing cells under glucose starvation after knocking down of SIK2. Our data showed knocking down of SIK2 in U251B55gamma cells significantly re-sensitized cells to glucose depletion in both U251B55gamma and U138B55gamma cells (Figure 5B), revealing that the association between B55gamma and SIK2 was required for the tolerance of glucose starvation.

## Discussions

As we discussed above, PPP2R2C encodes a gamma isoform of the B regulatory subunit subfamily-B55 gamma among the four unrelated B subunit families. Currently, the precise functions of B55gamma are still unclear. It has been reported that B55gamma had a unique expression pattern in mouse brain which suggested it correlated to intellectual disability [20]. Another study reported that B55gamma was an inhibitor of c-Jun NH2-terminal kinase (JNK) activation by UV irradiation through the association with c-SRC [7]. A recent paper reported the loss of B55gamma promotes androgen ligand depletion-resistant prostate cancer growth, which was independent of AR-mediated transcriptional programs indicating B55gamma might be a tumor suppressor gene [8]. In this project, we reported overexpression of B55gamma in glioma cells suppressed the phosphorylation of S6K, indicating B55gamma might be a putative tumor suppressor gene.

The metabolic properties of cancer cells are different from those of normal cells. Cancer cells are more dependent on aerobic glycolysis, fatty acid synthesis and glutaminolysis for proliferation [10]. Multiple oncogenes or tumor suppressors can regulate cancer cells glucose metabolism. Recently, P53 has been reported to regulate aerobic respiration at the glycolytic and oxidative phosphorylation (OXPHOS) steps via transcriptional regulation of its downstream genes [13]. Since B55gamma is a potential tumor suppressor gene, whether B55gamma participates in the regulatory pathway of glucose metabolism or not triggered us to establish this project. In this study, we reported a novel function of B55gamma in the regulation of glucose metabolism in Glioma cells. We stably transfected B55gamma in Glioma cells and noticed that B55gamma suppressed the cancer cell glucose uptake and lactate product. Our data demonstrated under glucose starvation, exogenous overexpression of B55gamma rendered Glioma cells adaptive to glucose starvation compared with control cells.

We reported that B55gamma inhibited the activity of S6K through the association with SIK2. It is interesting since SIK2 belongs to the AMPK family and the function of SIK2 has not been deeply elucidated. As well-studied, AMPK is a central metabolic switch found in all eukaryotes that governs glucose and lipid metabolism in response to alterations in nutrients and intracellular energy levels. It has been reported under nutrition starvation, AMPK was activated to suppress mTOR-dependent transcriptional regulators to keep tumor cell survival [21]. A recent study described a putative mechanism for the B55gamma-mediated de-phosphorylation of S6K by PP2A [22]. From their results, B55gamma does not bind with S6K, but promotes the formation of the PP2A complex with the PP2A-C subunit to further enhance the binding of the catalytic subunit of PP2A with S6K. Their data revealed that B55gamma down-regulates the S6K pathway might through the PP2A-S6K interaction, but not the PP2A-mTOR interaction, providing a mechanism for the dephosphorylation of S6K by overexpression of B55gamma. Moreover, they did not show any change on the activity of 4EBP1 by overexpression of B55gamma, the similar results as us. Thus, both of these two studies showed a consistently regulatory relationship between B55gamma and S6K, but not 4EBP1, indicating B55gamma indirectly targeting on S6K which is a downstream effector of mTOR, but not mTOR itself. Our data showed B55gamma associates with and stabilizes SIK2 which was indispensably participated in the inhibition of S6K-dependent growth regulation to keep tumor cell survival under glucose depletion. However, the detailed mechanisms still need further investigation. We are constructing antibodies which specific recognize phosphorylation statues of SIK2 since there is no commercial phosphor-SIK2 antibody available. Our next project will focus on how B55gamma regulates SIK2 through the association and how to target B55gamma-SIK2-S6K pathway to develop therapeutic strategies for the treatment of human brain tumor patients.
